# Green tea leaf powder prevents dyslipidemia in high-fat diet-fed mice by modulating gut microbiota

**DOI:** 10.29219/fnr.v64.3672

**Published:** 2020-11-13

**Authors:** Jin Wang, Ping Li, Shuang Liu, Bowei Zhang, Yaozhong Hu, Hui Ma, Shuo Wang

**Affiliations:** 1 Tianjin Key Laboratory of Food Science and Health, School of Medicine, Nankai University, Tianjin, China; 2 State Key Laboratory of Food Nutrition and Safety, Tianjin University of Science and Technology, Tianjin, China

**Keywords:** green tea leaf powder, dyslipidemia, gut microbiota, satiety, inflammation

## Abstract

**Background:**

In the past, most researchers paid more attention to the biological activity of tea infusion and tea polyphenols; however, the prebiotic role of tea leaf powder is still unknown. Green tea leaf powder is rich in dietary fiber and is suggested to be beneficial for human health. Only limited studies have looked at the effects of tea leaf powder (which mainly contains tea dietary fiber) on gut microbiota and health.

**Objective:**

The purpose of our study was to determine the effects of green tea leaf powder in preventing hyperlipidemia and to understand its potential lipid-lowering mechanism.

**Design:**

Mice in three treatment groups were fed high-fat diets (HFDs) by administering either 0.5, 1.0, or 2.0 g/kg•d dietary fiber-enriched green tea leaf powder of low, medium, or high, respectively, for 12 weeks. Serum biochemical analyses and mRNA gene expression levels of related energy and lipid metabolism biomarkers from the liver were investigated. In addition, 16S rRNA cecal microbiota and fecal short chain fatty acids (SCFAs) were tested.

**Results:**

Green tea leaf powder reduced body weight and total cholesterol of HFD-fed mice in a dose-dependent manner. Green tea leaf powder also increased satiety hormone secretion and reduced systemic inflammation of HFD-fed mice. Real-time polymerase chain reaction (PCR) analyses reconfirmed that green tea leaf powder prevented dyslipidemia by enhancing hepatic mRNA expression levels of peroxisome proliferator-activated receptor alpha, cholesterol 7α-hydroxylase, and Adenosine triphosphate (ATP)-binding cassette transporter A1 and decreasing the expression of fatty acid synthase, sterol regulatory element-binding protein 1c, and liver X receptor. Green tea leaf powder promoted the growth of *Blautia*, *Oscillibacter*, *Ruminiclostridium*, *Alloprevotella*, and *Butyrivibrio* and inhibited the growth of *Erysipelatoclostridium*, *Desulfovibrio*, and Candidatus_*Saccharimonas* in the cecum of HFD-fed mice.

**Conclusion:**

In summary, our results indicate that green tea leaf powder improves lipid metabolism of HFD-fed mice in a dose-dependent manner. The potential mechanism involves a synergistic role in reprogramming gut microbiota, increasing satiety hormone secretion, and reducing systemic inflammation.

## Popular scientific summary

- Green tea leaf powder improves lipid metabolism of high-fat diet-fed mice in a dose-dependent manner.
- The potential mechanism involves a synergistic role in reprogramming gut microbiota, increasing satiety hormone secretion, and reducing systemic inflammation.

Consuming a high-fat diet (HFD) over a prolonged period seriously affects human health and leads to metabolic disorders and chronic diseases. In particular, excessive fat intake increases the risk of dyslipidemia, which is the primary reason for cardiovascular disease around the world. As an alternative to pharmacological medicine, dietary intervention such as prebiotics has been used to prevent or mitigate chronic metabolic disease. Increasing evidence supports that consuming foods rich in dietary fiber can reduce the incidence of metabolic disease (1). The recommended daily intakes of dietary fiber are 25 and 38 g for adult women and men, respectively (2). However, dietary fiber intake for most of the people across the world does not meet this recommendation.

Considerable research has shown that dietary fiber improves health. Its intake is known to increase satiety, affect energy intake, reduce weight, and control blood glucose and lipid metabolism (3). The interaction between dietary fiber and gut microbiota can have a major impact on host metabolism. Since dietary fiber cannot be digested by human enzymes, it directly reaches the large intestine. Dietary fiber can promote beneficial microbiota growth and inhibit harmful microbiota development, which help maintain the balance of intestinal flora. The major products of microbial fermentation with dietary fiber are SCFAs, including acetate, propionate, and butyrate. SCFAs serve as an energy resource for colonocytes and can also activate G-protein-coupled-receptors, which contribute to the modulation of host energy metabolism (4). In addition, SCFAs are known as histone deacetylase inhibitors, which result in anti-inflammatory effects (4).

Tea is one of the three most widely consumed beverages in the world and has high nutritional value and health function. Tea contains several nutritional and functional ingredients, including polyphenols and dietary fiber. In the past, most researchers have paid more attention to the biological activity of tea infusion and tea polyphenols; however, the prebiotic role of tea leaf powder, which is rich in dietary fiber, is still unknown. The traditional way of drinking tea results in almost all the dietary fiber being discarded as waste residue. Few studies have paid attention to the effects of tea leaf powder, which mainly includes tea dietary fiber, on gut microbiota and health. A previous article showed that tea dietary fiber could improve serum and hepatic lipid metabolism; however, whether the lipid-lowering effects were due to the modulation of gut microbiota, was not studied (5). Another article has also shown that green tea leaf powder significantly reduced the body fat content, hepatic triacylglycerol, and cholesterol accumulation in high-fat fed C57BL/6J mice (6). In addition, these effects of improving lipid metabolism are due to the increase of *Akkermansia* amounts in the small intestine using viable count, T-RFLP, and qPCR. However, high percentage of the gut microbiota has not been cultured. In addition, Terminal restriction fragment length polymorphism (T-RFLP) and qPCR require fluorescently labeled primer, have been proven to have low sensitivity, and being capable of detecting only abundant microbial taxa (>1%). Therefore, results of green tea leaf powder on total bacterial composition and diversity were lacking in HFD mice model. Green tea leaf powder is increasingly included as supplementary ingredients in foods, such as special drinks, biscuits, bread, desserts, etc. The comprehensive utilization of tea leaf powder can greatly improve the value of the tea industry, which is conducive to the development of the food industry and agriculture.

The purpose of our study was to determine the effects of green tea leaf powder in the prevention of hyperlipidemia and to understand its potential lipid-lowering mechanism. Our results suggest that green tea leaf powder could prevent HFD-induced dyslipidemia synergistically by modulating gut microbiota, increasing satiety hormone secretion, and reducing inflammation.

## Materials and methods

### Preparation of green tea leaf powder and analyses of its chemical composition and physiochemical properties

Green tea leaf powder was manufactured from green tea using hot water extraction, dehydration, and drying. Green tea raw materials were mixed in 90°C water for 30 min. The mixture was then dehydrated using a screw extrusion machine for 60 sec with a pressure of 0.5–1.2 MPa. Drying temperature was 100–130°C for 3–5 h. Next, the dehydrated green tea leaf was ground to powder with particles size 80–1,500 mesh and stored at 4°C prior to analysis.

Water, ash, protein, soluble, insoluble, and total fiber contents, and physicochemical properties including water-holding, swelling, and oil-holding capacity were determined as described previously (7). Cellulose, hemicellulose, lignin, and pectin were also determined as described previously (8). Tea polyphenol was determined according to GB/T 31740.2-2015.

### Animal experiments

All animal experiments were approved by the Institutional Animal Care and Use Committee of Nankai University. Eight-week-old male C57BL/6J mice were obtained from SPF Biotechnology Co., Ltd (Beijing, China) and housed at a constant temperature of 25°C and relative humidity of 50% in 12 h light–dark cycles. The mice had free access to water and food. After the acclimatization period of 1 week, the mice were randomly divided into five groups (seven mice per group). Negative control (NC) group were fed with a normal diet (AIN-93). Model control (MC) group were fed with an HFD (78.8% basal feed, 1% cholesterol, 10% yolk powder, 10% lard, and 0.2% cholate). The other three groups were fed with an HFD, but were also administered either 0.5 g/kg•d dietary fiber-enriched green tea leaf powder-low (DFL),1.0 g/kg•d dietary fiber-enriched green tea leaf powder-medium (DFM), or 2.0 g/kg•d dietary fiber-enriched green tea leaf powder-high (DFH) for 12 weeks by intragastric gavage. Body weight and food intake were assessed once a week. Feces of mice were collected before the end of the experiment for measuring SCFAs. SCFAs in feces were determined as previously described (9). Mice were sacrificed by anesthesia with somnopentyl after 12 h fasting. Blood, liver, and cecum contents were collected. Serum was centrifuged at 4,200 rpm for 15 min and stored at −20°C. The collected samples were immediately frozen in liquid nitrogen and stored at −80°C for further research.

### Serum biochemical analysis

The levels of total triglyceride (TG), total cholesterol (TC), high-density lipoprotein-cholesterol (HDL-C), and low-density lipoprotein-cholesterol (LDL-C) were determined using commercial kits (Nanjing Jiancheng Bioengineering Institute, Nanjing, China). Glucose and insulin concentrations were also determined using commercial kits (BioSino Biotechnology and Science Incorporation, Beijing, China).

Peptide YY (PYY) and glucagon-like peptide-1 (GLP-1) levels were determined as previously described (10). Leptin and adiponectin levels were determined by using ELISA kits (Bio-Swamp Life Science, Wuhan, China). Tumor necrosis factor-alpha (TNF-α), interleukin-6 (IL-6), and lipopolysaccharides (LPS) were detected using commercial kits (Sinouk Institute of Biological Technology, Beijing, China).

### Real-time quantitative PCR

Total RNA was extracted from the liver using RNeasy Mini Kit (Qiagen, Hilden, Germany) and quantified using a Nanophotometer (Implen, Germany) and a agarose gel electrophoresis. cDNA was synthesized using LunaScript^TM^ SuperMix Kit (NEB, MA, USA). Real-time qPCR was performed using the CFX Connect Real-Time System (BIO-RAD, USA). The primers used are listed in Table 1. The PCR procedure was as follows: initial denaturation at 95°C for 60 sec, 40 cycles at 95°C for 15 sec, and 60°C for 30 sec. Each gene expression was normalized to the reference β-actin gene.

**Table 1:** Primer sequences used for real-time qPCR

Gene	5’ Primer	3’ Primer
PPARα	TGCAGCCTCAGCCAAGTTGAA	TCCCGAACTTGACCAGCCA
SREBP1c	CTGGTGAGTGGAGGGACCAT	GACCGGTAGCGCTTCTCAAT
FAS	GAGGGTGTGCCATTCTGTCA	GCTATTCTCTACCGCTGGGG
LXR	AATGAAGCTGGTGAGCCTCC	CCATGTGGCCAACACAAAGG
CYP7A1	CATAGCCAACTTGCCGCAG	AGACAAAGCACTTGCCCTTC
ABCA1	TCCGTTGGCTTTCTCAGTCC	TTTGTTGTTGTTTTGTGGCCT
TLR4	GGAAGACAAAAGAAAGACAGCCC	TGGGGAGATTCTTGATCTGCT
β-Actin	ACAGCAGTTGGTTGGAGCAA	ACGCGACCATCCTCCTCTTA

PPARα, peroxisome proliferator-activated receptor alpha; SREBP1c, sterol regulatory element-binding protein 1c; FAS, fatty acid synthase; LXR, liver X receptor; CYP7A1, cholesterol 7α-hydroxylase; ABCA1, ATP-binding cassette transporter A1; TLR4, toll-like receptor 4.

### Cecal microbiota analyses using 16S rDNA gene sequencing

Total genomic DNA, of the contents of the cecum, was extracted using QIAamp DNA Stool Minikit (Qiagen, Hilden, Germany). V3–V4 region of 16S rRNA gene was amplified using forward primer 341F (5’-CCTAYGGGRBGCASCAG-3’) and reverse primer 806R (5’-GGACTACNNGGGTATCTAAT-3’). PCR products were purified by GeneJETTM gel extraction kit (Thermo Scientific, MA, USA). Sequencing was performed on an Ion S5^TM^ XL platform (Novogene Co. Ltd, Beijing, China).

Sequence analyses were done with Uparse software, and sequences with more than 97% similarity were assigned to an operational taxonomic unit. The Silva Database (Version 132) was used to categorize the taxonomic information (phylum, class, order, family, and genus). Alpha diversity (observed species, Chao1, Shannon, and PD_whole_tree) was analyzed. Unweighted unifrac, for principal coordinate analysis (PCoA), was used to investigate the differences between groups. Phylogenetic investigation of communities by reconstruction of unobserved states (PICRUSt) analysis was used to predict gut microbiota function, and the Kyoto encyclopedia of genes and genomes (KEGG) database was used for reference genomes. Linear discriminant analysis effect size (LEfSe) analysis was conducted to find representative microbiota between groups.

### Statistics

Data are expressed as mean ± SEM. Differences with *P*-value < 0.05 were deemed to be significant. Analysis was performed using SPSS software.

## Results

### Chemical composition and physiochemical properties of green tea leaf powder

Green tea leaf powder consisted of 61.15 ± 1.24% total dietary fiber (54.62 ± 1.10% insoluble and 6.53 ± 0.19% soluble) (Table 2). Insoluble dietary fiber mainly contains 20.33 ± 1.39% cellulose, 10.91 ± 2.02% hemicellulose, and 12.77 ± 0.38% lignin. Soluble dietary fiber mainly contains 4.56 ± 0.10% pectin. In addition, green tea leaf powder exhibited higher water, oil-holding capacity, and swelling capacity.

**Table 2:** Chemical composition and physiochemical properties of green tea leaf powder

Chemical composition and physiochemical properties	Contents (average ± SD)
*Chemical composition*	
Water content	10.91 ± 0.12%
Ash	2.89 ± 0.34%
Protein	20.61 ± 0.23%
Fat	0.87 ± 0.04%
Tea polyphenol	6.20 ± 0.07%
Total dietary fiber	61.15 ± 1.24%
Soluble dietary fiber	6.53 ± 0.19%
Insoluble dietary fiber	54.62 ± 1.10%
Cellulose	20.33 ± 1.39%
Hemicellulose	10.91 ± 2.02%
Lignin	12.77 ± 0.38%
Pectin	4.56 ± 0.10%
*Physiochemical properties*	
Water-holding capacity	3.49 ± 0.05 g/g
Swelling capacity	2.53 ± 0.03 mL/g
Oil-holding capacity	1.85 ± 0.21 g/g

### Effect of green tea leaf powder on body and liver weight

**Fig. 1:**
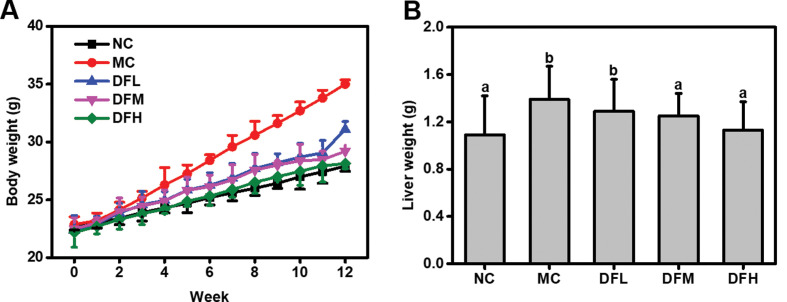
Green tea leaf powder reduces the body weight (A) and liver weight (B) of HFD-fed mice. Values were measured in mice from NC, MC, DFL, DFM, and DFH groups (*n* = 7).

After 4 weeks, body weight of the MC group mice increased by 15.28% of the initial value, while the NC group mice only gained 6.73% (Fig. 1). The mice fed with green tea leaf powder showed a reduction in body weight compared to the MC group in a dose-dependent manner. In particular, a high dosage of green tea leaf powder was able to significantly reduce the body weight of HFD-fed mice (*P* < 0.05). In addition, green tea leaf powder also reduced live weight of HFD-fed mice.

### Effect of green tea leaf powder on serum lipid and glucose levels

**Fig. 2:**
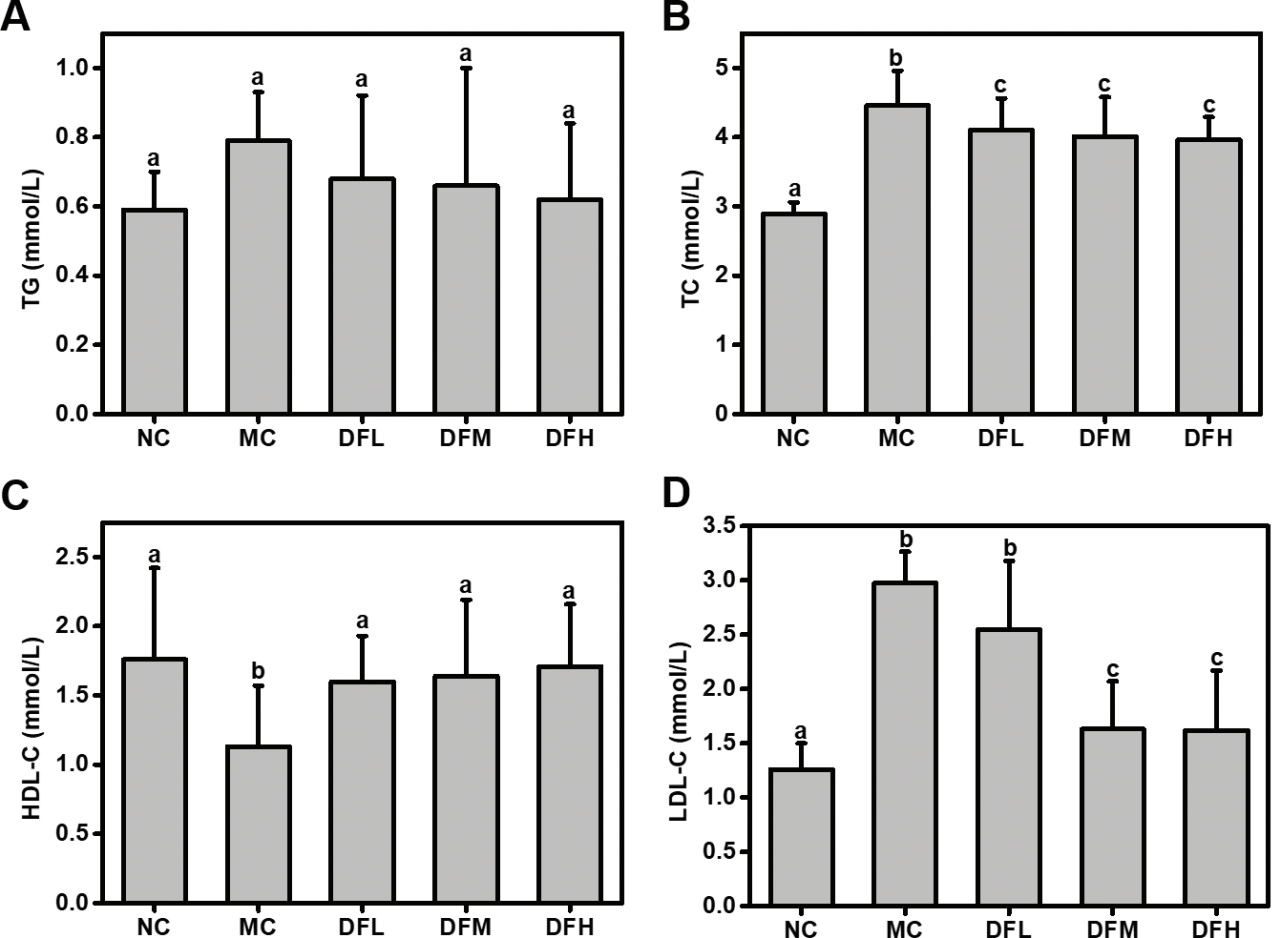
Effect of green tea leaf powder on the lipid metabolism. (A) TG, (B) TC, (C) HDL-C, and (D) LDL-C (*n* = 7). Significant differences (*P* < 0.05) are indicated using different letters (a, b, c).

Green tea leaf powder significantly reduced TC and LDL-C levels, and significantly increased HDL-C levels in a dose-dependent manner (*P* < 0.05) (Fig. 2). In particular, when compared to the MC group, a high dosage of green tea leaf powder significantly increased HDL-C level (*P* < 0.05). We also found a reduction in TG; however, the difference was not statistically significant.

**Fig. 3:**
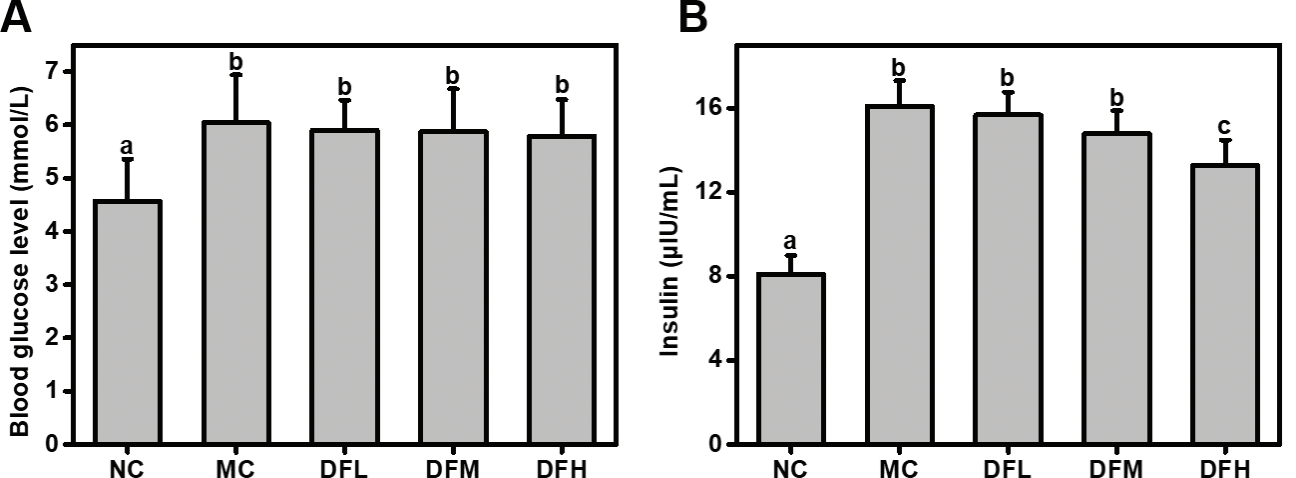
Effect of green tea leaf powder on glucose metabolism parameters. (A) Blood glucose levels and (B) insulin (*n* = 7). Significant differences (*P* < 0.05) are indicated using different letters (a, b, c).

A high dosage of green tea leaf powder significantly reduced insulin levels of HFD-fed mice (*P* < 0.05) (Fig. 3). Green tea leaf powder also slightly reduced fasting blood glucose level; however, this difference was not statistically significant.

### Effect of green tea leaf powder on body metabolism and energy balance

**Fig. 4:**
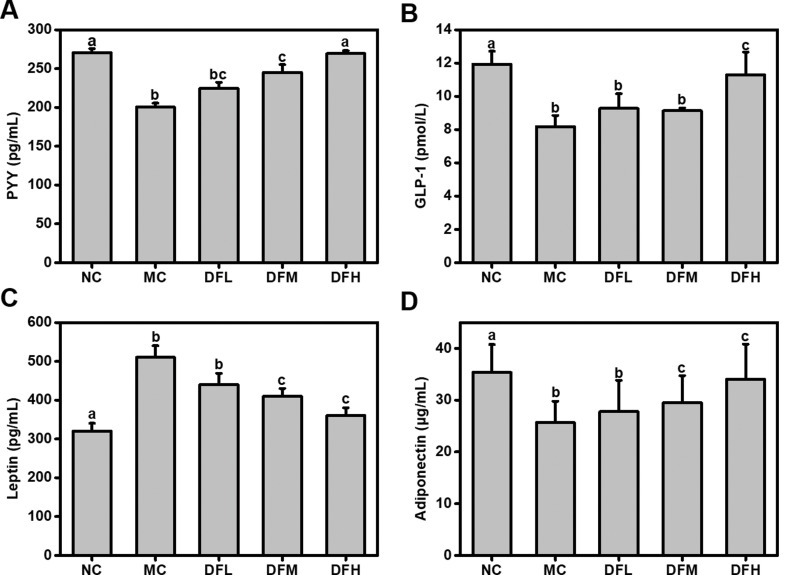
Effect of green tea leaf powder on serum hormone concentrations. (A) PYY, (B) GLP-1, (C) leptin, and (D) adiponectin (*n* = 7). Significant differences (*P* < 0.05) are indicated using different letters (a, b, c).

Green tea leaf powder significantly increased serum levels of the satiety hormones PYY and GLP-1 in a dose-dependent manner (Fig. 4). In particular, when compared to the MC group, a high dosage of green tea leaf powder significantly increased PYY and GLP-1 levels (*P* < 0.05). This shows that green tea leaf powder increased satiety hormone secretion and decreased appetite. In addition, a high dosage of green tea leaf powder could significantly increase serum adiponectin levels of HFD-fed mice (*P* < 0.05). Serum leptin levels were significantly increased in the MC group compared to the NC group, while the green tea leaf powder administration ameliorated this change.

### Effect of green tea leaf powder on systemic inflammation

**Fig. 5:**
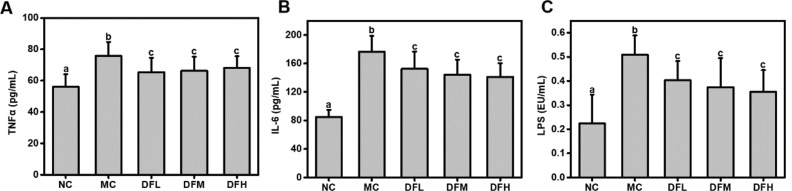
Effect of green tea leaf powder on systemic inflammation. (A) TNF-α, (B) IL-6, and (C) LPS (*n* = 7). Significant differences (*P* < 0.05) are indicated using different letters (a, b, c).

We observed higher levels of TNF-α, IL-6, and LPS in the serum of the MC group compared to the NC group (Fig. 5). Green tea leaf powder was able to significantly reduce serum TNF-α, IL-6, and LPS levels of HFD-mice (*P* < 0.05). However, no significant differences in levels were observed between the groups fed on various dosages of green tea leaf powder. Thus, green tea leaf powder could reduce metabolic endotoxemia and significantly alleviated systemic inflammation.

### Effect of green tea leaf powder on lipid metabolism-related mRNA gene expression

**Fig. 6:**
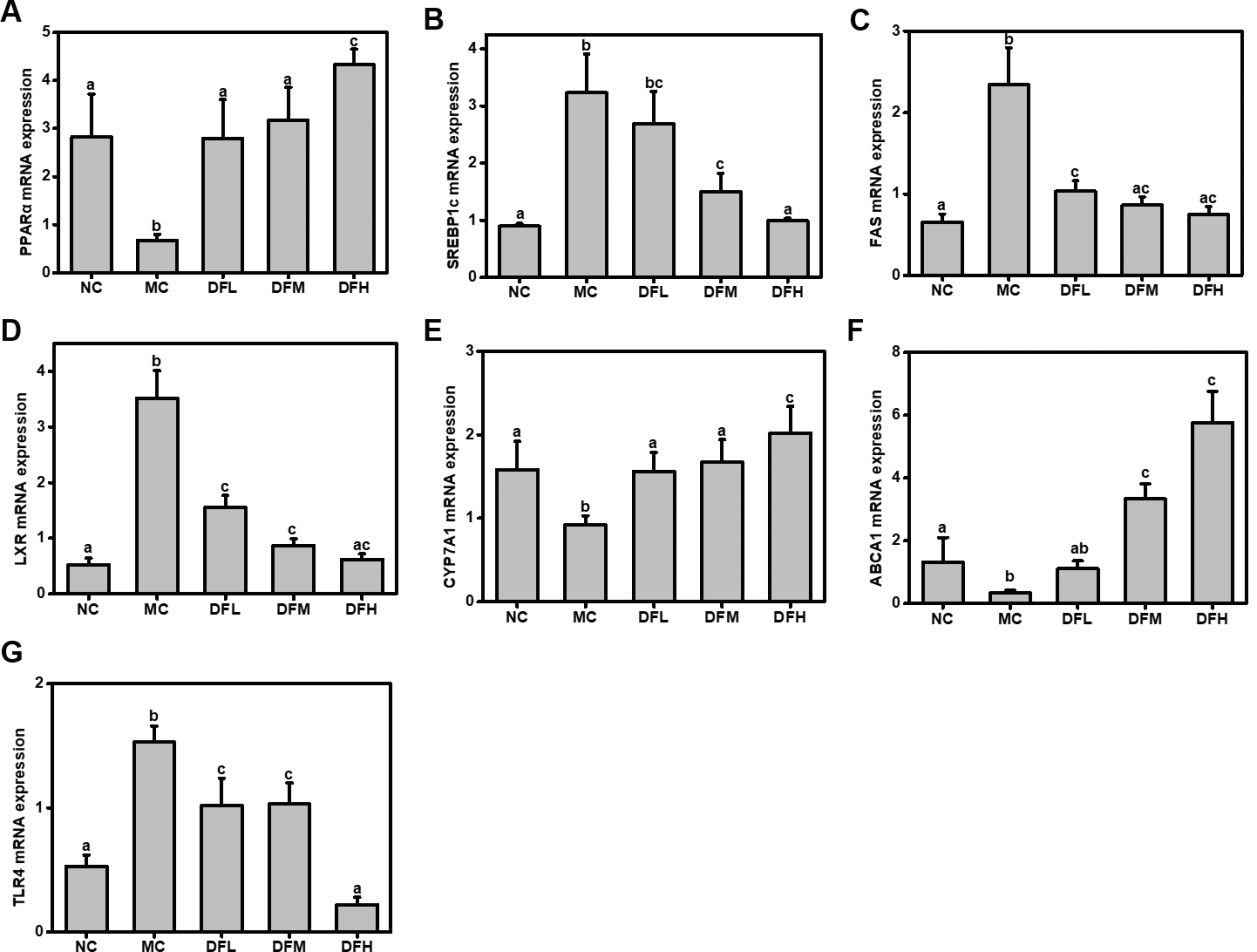
Effect of green tea leaf powder on mRNA expression levels of hepatic metabolic regulators. (A) PPARα, (B) SREBP1c, (C) FAS, (D) LXR, (E) CYP7A1, (F) ABCA1, and (G) TLR4 (*n* = 7). Significant differences (*P* < 0.05) are indicated using different letters (a, b, c).

Real-time PCR was performed to quantify the mRNA expression levels of genes that are biomarkers for energy and lipid metabolism. Compared to the NC group, the MC group had higher expression levels of lipid synthesis genes, including sterol regulatory element-binding protein 1c (SREBP1c), fatty acid synthase (FAS), and liver X receptor (LXR) and decreased the expression levels of PPARα, CYP7A1, and ABCA1 (Fig. 6). In contrast to the MC group, mice fed with green tea leaf powder showed significant (*P* < 0.05) dose-dependent increases in mRNA expression levels of PPARα, CYP7A1, and ABCA1. On the other hand, green tea leaf powder significantly suppressed mRNA expression of SREBP1c, FAS, LXR, and TLR4 (*P* < 0.05).

### Effect of green tea leaf powder on gut microbiota and SCFAs production

#### Richness and diversity analysis of gut microbiota

To assess the gut microbial community structure, richness and diversity were calculated using number of observed species, Chao1 estimator, Shannon index, and PD_whole_tree index (Table 3). With an increase in green tea leaf powder concentration, richness (observed species and estimated Chao1) was increased from 471 ± 49 and 582.7 ± 27.8 to 549 ± 44 and 679.4 ± 42.5, respectively. In contrast, the NC and MC groups had relatively low microbial community richness (observed species were 437 ± 31 and 398 ± 22, and Chao1 index was 540.3 ± 19.2 and 502.9 ± 27.0) (*P* < 0.05). Similarly, green tea leaf powder group had high microbial diversity (Shannon index varied from 5.2 ± 0.9 to 6.1 ± 0.7, and PD_whole_tree index between 29.6 ± 7.1 and 38.1 ± 6.9) compared to the NC and MC groups (Shannon index was 4.9 ± 0.8 and 4.4 ± 1.1, and PD_whole_tree index was 28.4 ± 6.6 and 25.3 ± 5.4) (*P* < 0.05).

**Table 3:** Alpha diversity estimates of gut microbiota associated with mice from NC, MC, DFL, DFM, and DFH groups (*n* = 7)

Groups	Observed species	Chao1	Shannon	PD_whole_tree
NC	437 ± 31	540.3 ± 19.2	4.9 ± 0.8	28.4 ± 6.6
MC	398 ± 22	502.9 ± 27.0	4.4 ± 1.1	25.3 ± 5.4
DFL	471 ± 49	582.7 ± 27.8	5.2 ± 0.9	29.6 ± 7.1
DFM	504 ± 37	614.6 ± 31.3	5.6 ± 0.5	35.9 ± 5.3
DFH	549 ± 44	679.4 ± 42.5	6.1 ± 0.7	38.1 ± 6.9

DFL, dietary fiber-enriched green tea leaf powder-low; DFM, dietary fiber-enriched green tea leaf powder-medium; DFH, dietary fiber-enriched green tea leaf powder-high; MC, model control; NC, negative control.

#### Green tea leaf powder modulates gut microbiota at different taxonomic levels

**Fig. 7:**
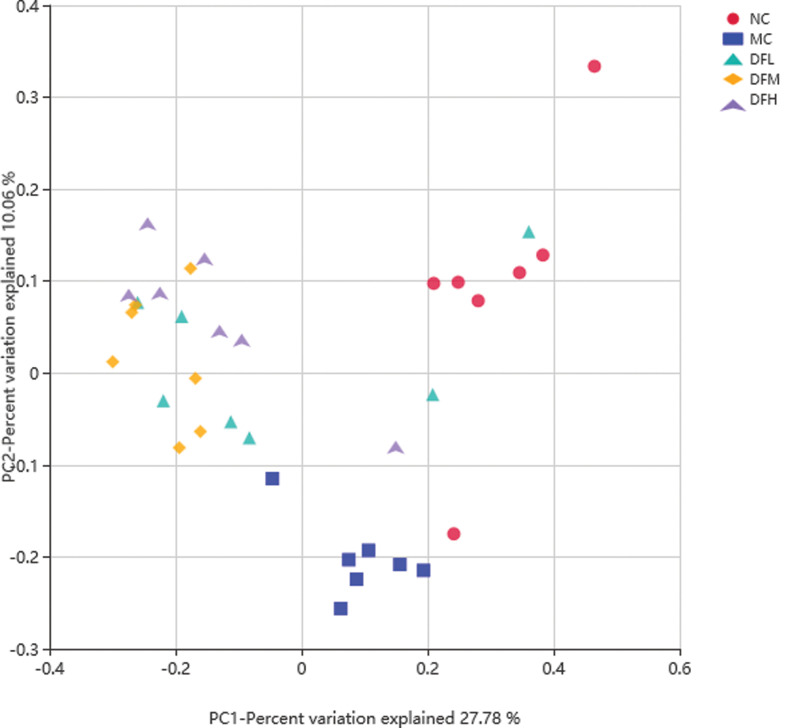
Beta diversity estimates for the gut microbiota from NC, MC, DFL, DFM, and DFH groups of mice (*n* = 7).

The phylogenetic differences between gut microbiota from NC, MC, DFL, DFM, and DFH groups of mice were assessed using PCoA analysis based on Bray-Curtis arithmetic (Fig. 7). Green tea leaf powder had a distinct microbiota composition that clustered separately from MC and NC groups.

To determine whether different concentrations of green tea leaf powder had an effect on the composition of cecal microbiota for HFD-fed mice, a taxon-dependent analysis was conducted.

**Fig. 8:**
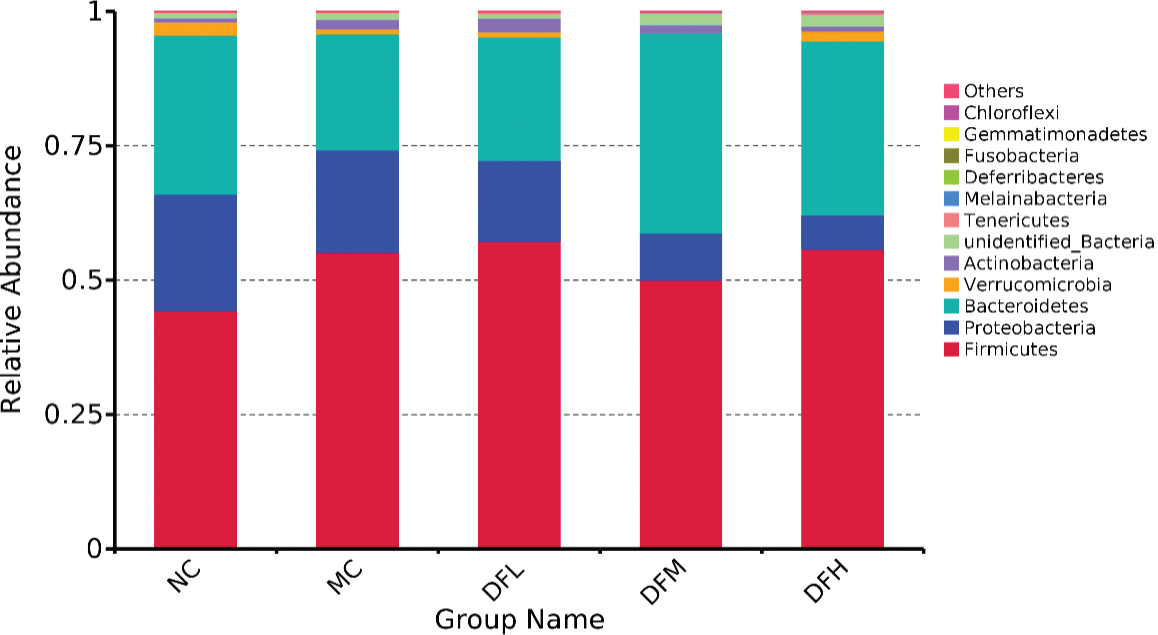
The average relative abundance of phyla in the cecum microbiota of mice fed with green tea leaf powder (*n* = 7).

At the phylum level (Fig. 8), an HFD showed a dramatic decrease in the relative abundance of *Bacteroidetes* and a trend toward an increase in the relative abundance of *Firmicutes* in the cecum compared to the NC group (*P* < 0.05). Green tea leaf powder significantly increased the relative abundance of *Bacteroidetes* and *Verrucomicrobia* and decreased the relative abundance of *Proteobacteria* and the ratio of *Firmicutes*/*Bacteroidetes* (F/B) (*P* < 0.05) in the cecum of HFD-mice. The numbers of *Actinobacteria* did not significantly change.

**Fig. 9:**
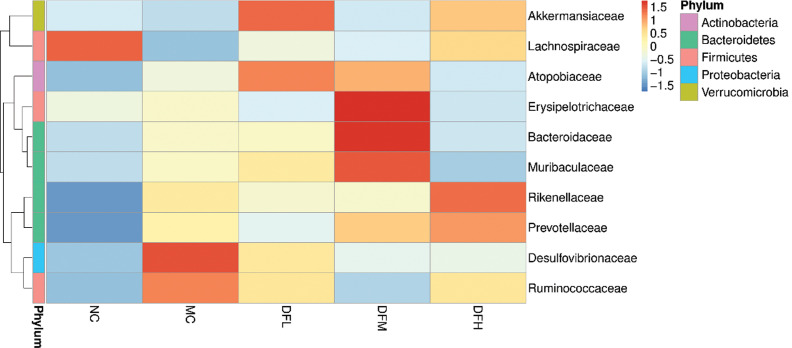
Heatmap of the average relative abundance of the 10 most abundant families in the cecum microbiota of mice fed with green tea leaf powder (*n* = 7).

The 10 most abundant families identified are shown in Fig. 9. Compared to the MC group, a high dosage of green tea leaf powder significantly increased the relative abundance of *Lachnospiraceae*, *Prevotellaceae*, and *Rikenellaceae* and significantly decreased the relative abundance of *Desulfovibrionaceae* and *Erysipelotrichaceae* in the cecum (*P* < 0.05). Green tea leaf powder also promoted the growth of *Akkermansiaceae*; however, we did not find any significant differences between dosage levels.

**Fig. 10:**
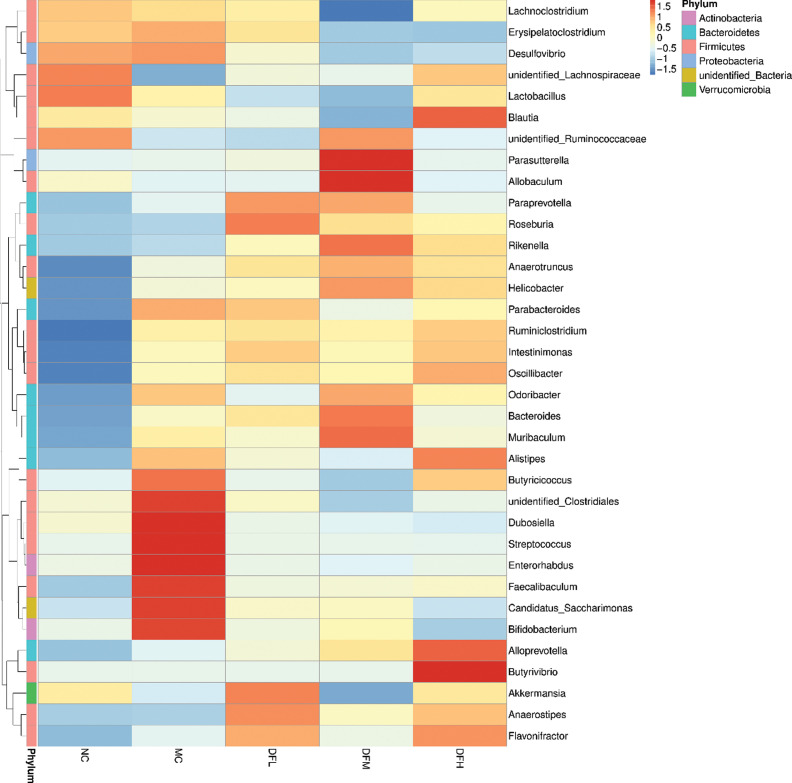
Heatmap of the average relative abundance of the 35 most abundant genera in the cecum microbiota of mice fed with green tea leaf powder (*n* = 7).

At the genus level (Fig. 10), compared to the MC group, a high dosage of green tea leaf powder significantly increased the relative abundance of *Blautia*, *Oscillibacter*, *Ruminiclostridium*, *Alloprevotella*, and *Butyrivibrio* and decreased the relative abundance of *Erysipelatoclostridium*, *Desulfovibrio*, and Candidatus_*Saccharimonas* (*P* < 0.05). A medium dosage of green tea leaf powder significantly increased the relative abundance of *Parasutterella*, *Allobaculum*, and *Anaerotruncus* (*P* < 0.05). A low dosage of green tea leaf powder significantly increased the relative abundance of *Roseburia*, *Akkermansia*, and *Anaerosipes* (*P* < 0.05).

**Fig. 11:**
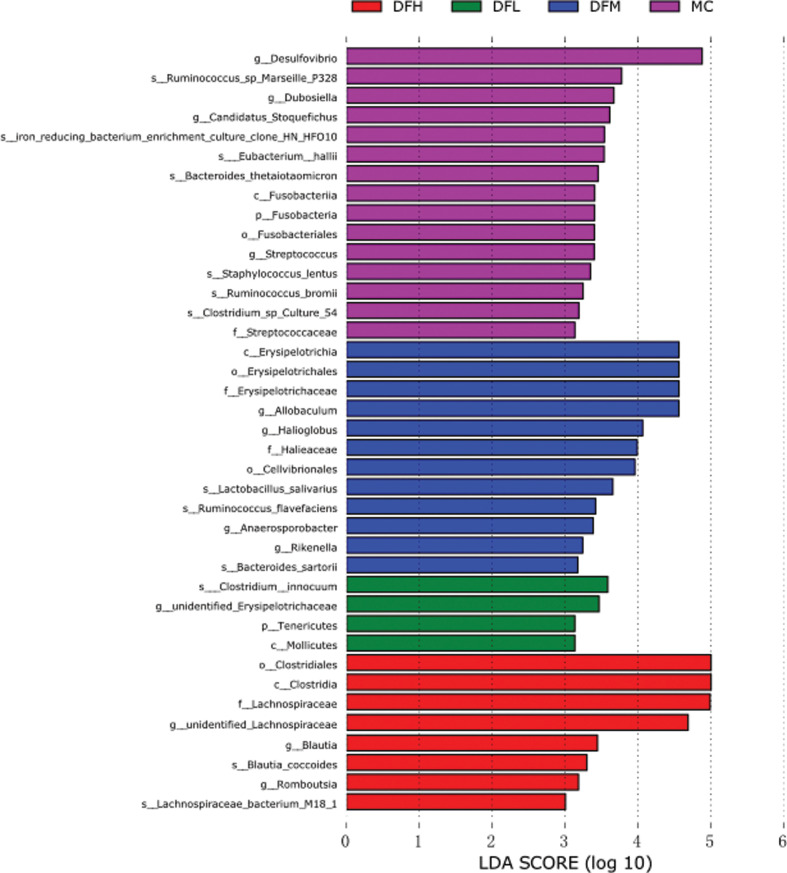
LEfSe analysis of gut microbiota between groups. Red shaded areas indicate DFH-enriched taxa, green shaded areas indicate DFL-enriched taxa, blue shaded areas indicate DFM-enriched taxa, and purple shaded areas indicate MC-enriched taxa (LDA > 3).

To identify the representative microbiota of the groups, gut microbiota from the different treatment groups were compared using LEfSe analysis. The LEfSe presented the enrichment of microbiota taxa, from phylum to species level. We found substantial differences between the different dosage levels of dietary treatments (Fig. 11). *Desulfovibrio* was significantly more abundant in the MC group. *Allobaculum* and *Erysipelotrichaceae* were more abundant in DFM group. *Lachnospiraceae* were more abundant in the DFH group.

#### Functional prediction of gut microbiota

The 12 KEGG biological pathways were significantly influenced in the gut microbiota with different dosages of green tea leaf powder (Table 4). Green tea leaf powder administration significantly increased fatty acid metabolism, butanoate metabolism, lipid metabolism, carbohydrate metabolism, energy metabolism, xenobiotics biodegradation and metabolism, cofactors and vitamins metabolism, cellular processes and signaling, and genetic information processing of gut microbiota. Meanwhile, green tea leaf powder administration significantly decreased metabolic disease, infectious diseases, and lipopolysaccharide biosynthesis of gut microbiota.

**Table 4:** List of significantly different KEGG pathways with greater abundance in MC, DFL, DFM, and DFH groups

KEGG pathway	Abundance mean, MC	Abundancemean, DFL	Abundance mean, DFM	Abundance mean, DFH
Fatty acid metabolism	22,120^a^	22,882^a^	27,899^b^	35,277^c^
Butanoate metabolism	32,107^a^	44,287^b^	56,333^b,c^	96,513^c^
Lipid metabolism	20,136^a^	22,457^a^	50,098^b^	76,501^c^
Carbohydrate metabolism	49,045^a^	89,764^b^	100,927^b,c^	152,826^c^
Energy metabolism	8,276^a^	8,255^a^	8,410^a^	9,745^b^
Metabolic diseases	2,750^a^	2,681^a^	1,863^b^	1,872^b^
Infectious diseases	2,654^a^	2,702^a^	1,893^b,c^	1,508^c^
Lipopolysaccharide biosynthesis	1,100^a^	923^b^	805^b^	789^c^
Xenobiotic biodegradation and metabolism	973^a^	1,028^b^	1,142^b^	1,047^b^
Cofactor and vitamin metabolism	1,340^a^	1,229^a^	1,341^a^	1,506^b^
Cellular processes and signaling	31,997^a^	34,901^b^	34,178^b^	33,793^b^
Genetic information processing	23,022^a^	22,015^a^	25,608^b^	24,435^b^

Significant differences (*P* < 0.05) are indicated using different letters (a, b, c).

DFL, dietary fiber-enriched green tea leaf powder-low; DFM, dietary fiber-enriched green tea leaf powder-medium; DFH, dietary fiber-enriched green tea leaf powder-high; MC, model control; NC, negative control.

#### Green tea leaf powder increases SCFAs production

Acetate, propionate, butyrate, and iso-butyrate levels in the feces were significantly reduced in the MC group compared to the NC group (*P* < 0.05) (Table 5). Compared to the MC group, green tea leaf powder administration increased acetate and butyrate levels in the feces in a dose-dependent manner. In particular, a high-dosage of green tea leaf powder significantly increased acetate and butyrate levels in the feces of HFD-mice (*P* < 0.05). The concentrations of other SCFAs did not show significant differences.

**Table 5:** Fecal SCFAs in C57BL/6J mice from NC, MC, DFL, DFM, and DFH groups (*n* = 7)

SCFAs (μmol/g)	NC	MC	DFL	DFM	DFH
Acetate	36.02 ± 2.37^a^	21.91 ± 3.32^b^	25.13 ± 3.81^b^	32.25 ± 5.06^a^	40.33 ± 6.48^a^
Propionate	1.23 ± 0.19^a^	0.89 ± 0.02^b^	0.95 ± 0.10^a,b^	1.28 ± 0.16^a,b^	1.43 ± 0.21^a,b^
Butyrate	4.39 ± 0.22^a^	3.51 ± 0.86^b^	3.98 ± 0.91^a,b^	5.02 ± 0.78^c^	6.02 ± 0.75^c^
Valerate	0.14 ± 0.02^a^	0.13 ± 0.06^a^	0.15 ± 0.03^a^	0.14 ± 0.04^a^	0.16 ± 0.05^a^
Iso-butyrate	0.68 ± 0.10^a^	0.15 ± 0.01^b^	0.26 ± 0.02^b^	0.33 ± 0.08^a,b^	0.47 ± 0.07^a^

Significant differences (*P* < 0.05) are indicated using different letters (a, b, c).

DFL, dietary fiber-enriched green tea leaf powder-low; DFM, dietary fiber-enriched green tea leaf powder-medium; DFH, dietary fiber-enriched green tea leaf powder-high; MC, model control; NC, negative control.

## Discussion

Green tea leaf powder had a preventive effect on dyslipidemia. From previous studies, the most effective active ingredient in green tea infusions is tea polyphenols, such as catechins. The tea polyphenols act as antioxidants and scavenging of free radicals for promoting health. For our study, the main bioactive ingredient of green tea leaf powder was dietary fiber. Green tea leaf powder could significantly ameliorate the HFD-induced dyslipidemia in a dose-dependent manner, which is possible by the synergistic modulation of gut microbiota, the action of gut satiety hormones, and enhancing anti-inflammatory cytokine production.

Satiety hormones can regulate appetite and play an important role in regulating body metabolism and energy balance. GLP-1 is a gut hormone from enteroendocrine L cells and it has many physiological functions, including promoting glucose-dependent insulin secretion as an incretin hormone, increasing pancreatic β-cell development, and inducing satiety via hypothalamus (11). PYY is also a gut anorexigenic hormone and acts by suppressing appetite in the brain, increasing gut transit rate, and reducing energy intake (12). In our study, food intake in the green tea leaf powder group was significantly lower than the MC group (data not shown), and the body weights of the mice in the green tea leaf powder group were significantly lower. Thus, the underlying mechanism which resulted in an improvement of dyslipidemia may be due to the reduction of food consumption and increase in satiety. High levels of leptin, an adipose tissue hormone, were observed in the MC group. This showed that the HFD-mice had developed leptin resistance, which probably resulted in insulin resistance and the development fatty liver disease (13). Green tea leaf powder reduced serum leptin levels in our study, and leptin resistance and sensitivity were improved. Increasing serum adiponectin levels using green tea leaf powder has been found to correlate with lipoprotein metabolism, resulting in an increase in HDL-C and a decrease in TG levels (14).

Diet and gut microbiota play an important role in low-grade inflammation characterized HFD-induced metabolic disease. Chronic and low-grade inflammation are characterized by elevated proinflammatory markers, including TNF-α and IL-6 (15). HFD-induced gut dysbiosis resulted in damage to gut integrity and the release of LPS from intestinal gram-negative bacteria into the blood, which could lead to chronic inflammation. Particularly, LPS could activate TLR4-induced insulin resistance (16). In our study, green tea leaf powder reduced metabolic endotoxemia and significantly alleviated systemic inflammation. Thus, another mechanism of improving dyslipidemia may be attributed to the alleviation of low-grade chronic inflammation.

It has been reported that the expression of genes related to hepatic fatty acid synthesis and oxidation could be altered by an HFD. Therefore, SREBP1c, FAS, PPARα, LXR, CYP7A1, and ABCA1 mRNA expression levels were evaluated in our study to investigate whether green tea leaf powder could influence the dyslipidemia in HFD-fed mice. SREBP1-c is a critical transcription factor, which participates in liver adipocyte differentiation and adipogenesis. Meanwhile, SREBP-1c was proved to be involved in hepatic cholesterol synthesis (17). Green tea leaf powder decreased SREBP-1c mRNA expression levels and could thus reduce fat and cholesterol synthesis. These results corresponded with lower TC levels of the green tea leaf powder group. FAS could enhance fatty acid synthesis and accumulate TG. LXR also plays an important role in lipogenesis. Green tea leaf powder could decrease the mRNA expression levels of FAS and LXR, thus it could reduce fatty acid synthesis. The mRNA levels of PPARα and CYP7A1 in the green tea leaf powder group were significantly higher than in the MC group. PPARα expression could promote fatty acid catabolism and reduce fat mass. CYP7A1 expression could improve hepatic steatosis and obesity by inhibiting hepatic lipogenesis (18). ABCA1 expression is vital for HDL-C formation and serves as a cellular efflux transporter of liver lipid and cholesterol. Therefore, the serum HDL-C level of the green tea leaf powder group is significantly higher than the MC group.

Increased ratios of F/B in the cecum are associated with obesity and metabolic disease. In our study, green tea leaf powder increased the relative abundance of *Bacteroides* and decreased the ratio of F/B. Thus, green tea leaf powder can potentially reduce weight and alleviate metabolic disease. *Verrucomicrobia* contains *Akkermansia*, a mucin-degrading microbiota, which was reported to play an important role in preventing obesity and metabolic disease (19).

*Lachnospiraceae* is involved in the production of butyrate (20). *Erysipelotrichaceae* is related to inflammation gastrointestinal diseases and is enriched in colorectal cancer (21). *Desulfovibrionaceae* is potentially an endotoxin LPS producer, which could induce a potent inflammation reaction (22). A lower abundance of the *Desulfovibrionaceae* was discovered in the green tea leaf powder group compared to the MC group, which may help in alleviating inflammation. Green tea leaf powder intervention increased *Prevotellaceae* and *Rikenellaceae*, which are hydrogen-producing bacteria and are similar to lactulose intervention (23). Hydrogen could ameliorate the symptoms of inflammatory bowel disease by reducing oxidative stress.

A high dosage of green tea leaf powder could significantly increase the relative abundance of *Blautia*, *Oscillibacter*, *Ruminiclostridium*, *Alloprevotella*, and *Butyrivibrio*. *Blautia* was reported to have the ability to improve glucose and lipid homeostasis (24). *Oscillibacter* is related to reduce body weight (25). *Ruminiclostridium* was reported to degrade cellulose and lignin in nature (26). *Alloprevotella*, an SCFA-producing bacteria, was negatively related to obesity and metabolic syndrome (27). *Butyrivibrio* is a well-known butyrate-producing bacteria (28), and our results also showed that a high dosage of green tea leaf powder significantly increased butyrate concentrations, whereas a high dosage of green tea leaf powder significantly decreased the abundance of *Erysipelatoclostridium*, *Desulfovibrio*, and Candidatus_*Saccharimonas*. *Erysipelatoclostridium* was considered an opportunistic pathogen in the gut (29). *Desulfovibrio* is a gram-negative bacteria and also a potential LPS producer (22). Candidatus_*Saccharimonas* was found in a high proportion of the feces of HFD-fed mice, which is consistent with our results (30). Thus, green tea leaf powder can decrease the relative abundance of pathogenic microbiota and improve the gut homeostasis.

## Conclusion

Our results show that green tea leaf powder supplementation in HFD-mice improves dyslipidemia in a dosedependent manner, which is possible by the synergistic modulation of gut microbiota, the action of gut satiety hormones, and enhancing anti-inflammatory cytokine production. These results imply that green tea leaf powder intervention may be useful in modulating some of the adverse effects that accompany HFD-induced dyslipidemia.
